# Analysis of blood coagulation in mice: pre-analytical conditions and evaluation of a home-made assay for thrombin-antithrombin complexes

**DOI:** 10.1186/1477-9560-3-12

**Published:** 2005-08-22

**Authors:** Dirkje W Sommeijer, René van Oerle, Pieter H Reitsma, Janneke J Timmerman, Joost CM Meijers, Henri MH Spronk, Hugo ten Cate

**Affiliations:** 1 Laboratory for Experimental Internal Medicine, Academic Medical Center of Amsterdam, Amsterdam, The Netherlands; 2 Department of Vascular Medicine, Academic Medical Centre of Amsterdam, Amsterdam, The Netherlands; 3 Laboratory for Clinical Thrombosis and Haemostasis, Department of Internal Medicine, and Cardiovascular Research Institute Maastricht, University of Maastricht, The Netherlands

## Abstract

**Background:**

The use of mouse models for the study of thrombotic disorders has gained increasing importance. Methods for measurement of coagulation activation in mice are, however, scarce. The primary aim of this study was to develop a specific mouse thrombin-antithrombin (TAT) ELISA for measurement of coagulation activation and to compare it with two commercially available assays for human TAT complexes. In addition, we aimed to improve methods for mouse plasma anticoagulation and preparation.

**Methods and results:**

First, for the measurement of TAT-complexes in plasma a mouse specific TAT-ELISA was developed using rabbit polyclonal antibodies raised against mouse thrombin and rat antithrombin, respectively. This ELISA detected an increase in TAT levels in a mouse model of endotoxemia. Two commercial human TAT ELISAs appeared to be less specific for mouse thrombin-rat antithrombin complexes.

Second, to prevent clotting of mouse blood sodium citrate was either mixed with blood during collection in a syringe or was injected intravenously immediately prior to blood collection. Intravenous sodium citrate completely inhibited blood coagulation resulting in plasma with consistently low TAT levels. Sodium citrate mixed with blood during collection resulted in increased TAT levels in 4 out of 16 plasma samples. Third, heparinase was added to plasma samples after in vivo injection of different heparin doses to test its neutralizing effect. Heparinase neutralized up to a 20 U of heparin/mouse and resulted in accurate APTT and factor VIII determinations.

**Conclusion:**

These procedures and reagents for plasma preparation and coagulation testing will improve studies on thrombotic disorders in mice.

## Background

Since the identification of genes encoding mouse clotting proteins and the creation of a growing number of transgenic mice, the use of mouse models for the study of thrombotic disorders has gained increasing interest [1]. While methodology for determining fibrin deposition in tissues [2] and quantification of thrombus formation [2,3] is readily available, optimal utilization of blood samples from mice remains a poorly explored area.

The availability of proper laboratory assays is essential in order to study coagulation activation in mice, comparably to humans. There is, however, a striking lack of analytical methods to test coagulation activation specifically in mice. For human studies, several commercial assays are available, including assays for thrombin-antithrombin (TAT) complexes to test *in vivo *coagulation activation [4,5]. When these assays, based on antibody detection of human protein epitopes, are applied on mouse plasma, it is likely that difficulties can arise depending on the degree of interspecies cross-reactivity, sensitivity, and specificity.

A practical issue of importance is that, traditionally, research on blood coagulation depends on determination of the activity of coagulation factors in a plasma sample. For this purpose specific anticoagulants, including sodium citrate or cocktails of substances need to be added to blood samples, in order to prevent *ex vivo *clotting. While even in humans the collection of proper plasma samples is not without practical problems, the collection of plasma from mice is a major technical challenge. As far as we know, there are no published procedures for anticoagulation that specifically address plasma sampling in mouse.

The use of mouse models enables investigations into the mechanism of coagulation activation in plasma as well as its consequences, i.e. fibrin deposition and pathological changes in organs, simultaneously. However, for proper tissue collection procedures such as heparinization are frequently required, which impairs most conventional tests of plasmatic coagulation. Human studies showed that addition of heparinase to plasma samples neutralized up to 2 U/ml heparin resulting in accurate factor VIII determinations [6]. In order to minimize interference of heparin we tested the effect of heparinase added to mouse plasma samples following *in vivo *heparinization.

In this study we developed a new mouse specific TAT ELISA and validated this method using a mouse model of endotoxemia, characterized by enhanced coagulation activation. Furthermore, we compared this new ELISA with two commercially available human immunoassays for measuring thrombin-antithrombin complexes in mouse plasma samples collected by different blood collection procedures.

## Methods

### Animals

All studies were performed in male mice of the C57Bl/6 strain (University of Maastricht or Academic Medical Center, Amsterdam), which were 8 weeks old and weighing approximately 20 grams at the start of the experiments. The animals were housed in normal cages in an environment with a 12 hour light-dark cycle, controlled temperature (20 ± 2°C) and humidity (50 ± 10%). Animals had free access to water and diets (Hope Farms, Woerden, The Netherlands). Endotoxemia studies were performed by i.p. injection of 2 mg lipopolysaccharide (LPS, E. coli, Sigma, St.Louis, MO) per kg. The Experimental Animal Ethics Committee of the University Maastricht and of the Academic Medical Center, Amsterdam approved all experimental protocols.

### Chemicals

All reagents were of analytical grade or better and were from commercial suppliers.

#### Development of TAT-ELISA

##### Rabbit immunization

Mouse thrombin (Sigma, St.Louis, MO, USA) and rat antithrombin (AT) (Sigma) were dissolved in PBS (150 mmol/L NaCl, 10 mmol/L sodium phosphate, pH 7.4), to a concentration of 200 and 140 μg/ml, respectively. Fifteen-week old New Zealand White rabbits, weighing approximately 3 kilograms were obtained from Harlan (Cambridge, UK). Immediately before immunization with thrombin and AT, pre-immune sera were collected. Thereafter, each rabbit was immunized with a mixture of 500 μl Freund's Complete Adjuvant (Difco, Detroit, MI, USA) and 500 μl protein solution. For each protein, two rabbits were injected twice s.c. on the back and twice i.m. in the hind legs. The rabbits were boosted at nine and 15 weeks after the first immunization with thrombin and AT in combination with Freund's Incomplete Adjuvant (Difco). During the first three months test samples of approximately five ml blood were obtained regularly and thereafter samples of approximately 50 ml were obtained monthly from each rabbit.

##### Reactivity of rabbit antisera for TAT ELISA

MaxiSorp plates (Nunc A/S, Roskilde, Denmark) were coated overnight at 4°C with 100 μl thrombin or AT in a concentration of 5 μg/ml diluted in coating buffer (70 mM Na_2_CO_3_, 30 mM NaHCO_3_, pH 9). Subsequently, the plates were washed three times with PBS, 0.05 %(v/v) Tween (PBS/Tween) and incubated with 100 μl rabbit sera diluted in PBS, 0.05 %(v/v) Tween, 1 %(v/v) FCS (PBS/Tween/FCS). The plates were incubated for 1 hour at room temperature and washed three times with PBS/Tween. Thereafter, the plates were incubated for 1 hour with 100 μl horseradish peroxidase (HRP)-conjugated swine anti-rabbit antibodies (DAKO A/S, Glostrup, Denmark) diluted 250-times in PBS/Tween/FCS. The plates were washed three times with PBS/Tween and developed with o-phenylenediamine (OPD, Sigma) for 30 minutes. The reaction was stopped with 100 μl 1 M H_2_SO_4 _and the optical density (OD) was determined at 490 and 650 nm. Calculations were performed using the SOFTmax software from Molecular Devices Corporation (Sunnyvale, CA, USA).

##### Purification of rabbit antibodies and conjugation to digoxigenin (DIG) for TAT ELISA

Three ml rabbit serum, 1/10 diluted with 200 mM sodium phosphate pH 7, was applied on a HiTrap Protein A column (Pharmacia LKB, Uppsala, Sweden). Before application of the sample the column was equilibrated with five column volumes 20 mM sodium phosphate, pH 7. After application of the sample the column was washed with 5 volumes 20 mM sodium phosphate and the rabbit IgG was eluted with 100 mM citric acid, pH 5. The pH of the IgG-elution sample was adjusted with 1 M Tris-HCl to pH 7 and dialyzed overnight at 4°C against PBS. The concentration of the purified IgG samples was adjusted to 1 mg/ml. One ml sample was incubated in a glass tube at RT under continuously shaking with 47 μl DIG-NHS uitleggen? ester (5 mg/ml, Roche Diagnostics). The samples were dialyzed overnight against PBS and frozen in aliquots at -20°C.

### In vitro generation of TAT-complexes

TAT-complexes were generated *in vitro *according to the method from Boisclair *et al*. (7). Briefly, 50 μg mouse thrombin and 140 μg rat antithrombin were dissolved in 330 μl TAT-buffer (50 mM Tris, 175 mM NaCl, 0.02% sodium azide, 0.1% PEG-6000, 4% BSA, pH 7.9) and incubated for 45 minutes at 37°C. The samples were aliquoted in 20 μl portions and frozen at -20°C.

### Western blot analysis

Western blot samples were prepared as follows: 5 μl 5x sample buffer (250 mM Tris-HCl pH 6.8, 10% SDS, 50% Glycerol) was added to 20 μl thrombin (50 μg/ml), 20 μl AT (50 μg/ml) or 20 μl *in vitro *TAT-complexes. The samples were prepared without addition of β-mercaptoethanol and were not denatured by heating because this results in aggregation of the *in vitro *TAT complexes in the PEG-6000 containing buffer. Plasma samples were prepared by combining 20 μl plasma, 20 μl 5x sample buffer and 60 μl H_2_O. Ten μl of each sample was separated on a 7.5% acrylamide gel and transferred by electroblotting to a nitrocellulose filter (Schleicher and Schuell, Dassel, Germany). After washing for 5 minutes with PBS/1%Tween and blocking for 1 hour at RT in PBS/Tween/5% (v/v) Milk the filters were incubated with 1/1000 diluted rabbit serum in PBS/Tween. After three washes of 10 minutes with PBS/Tween the blots were incubated for 1 hour at RT with HRP-conjugated swine anti-rabbit IgG diluted 250-fold in PBS/Tween. Finally, the blots were washed with PBS/Tween and H_2_O and incubated with a chemoluminescencent substrate (LumiLight, Roche Diagnostics, Mannheim, Germany). The pre-stained low range SDS PAGE markers (Bio-Rad Laboratories, Hercules, CA, USA) were used as molecular weight marker.

### Sandwich TAT-complex ELISA

TAT-levels in plasma were detected with the new mouse TAT ELISA as follows: MaxiSorp plates were coated overnight at 4°C with 100 μl purified anti-thrombin antibodies in a concentration of 1 μg/ml in coating buffer. Subsequently, the plates were washed three times with PBS/Tween and incubated with 50 μl standard or 4-fold diluted plasma samples in PBS/Tween/FCS for 1 hour on a shaker at room temperature. The plates were washed three times with PBS/Tween and incubated with 100 μl purified DIG-conjugated anti-AT antibodies in a concentration of 0.5 μg/ml for 1 hour at room temperature. The plates were washed three times with PBS/Tween and incubated for 1 hour at room temperature with 100 μl HRP-conjugated sheep F(ab)2 anti-DIG fragments (Roche Diagnostics) diluted in PBS/Tween. The plates were developed with OPD for 30 minutes and after termination of the reaction with 1 M H_2_SO_4 _the OD was determined at 490 and 650 nm. A standard for the mouse TAT ELISA was constructed with two-fold serial dilutions of mouse thrombin – rat antithrombin complexes (Sigma) in PBS/Tween/FCS buffer or mouse plasma.

#### Detection of TAT-complexes with three different ELISA's

The newly developed TAT-ELISA was compared with two commercially available ELISAs according to the manufacturer's instructions: Enzygnost^® ^TAT micro (DadeBehring BV, Leusden, The Netherlands) and TAT ELISA (Enzyme Research Laboratories (ERL), South Bend, IN, USA). For this purpose, *in vitro *TAT complexes were measured as standard and diluted in either buffer or normal mouse pool plasma. In addition, TAT-levels measured in plasma from control and LPS-treated (2 mg/kg i.p., 6 hours) mice, were compared.

#### Plasma collection procedures

##### Anti-coagulation with sodium citrate

Two methods of anticoagulation with sodium citrate were compared. In the first procedure, 900 μl blood was drawn from the vena cava into a syringe containing 100 μl 3.2% (w/v) sodium citrate. For the second approach 3.2% (w/v) sodium citrate in a total volume of body weight (gr) / 13 × 100 μl was i.v. administrated in the vena cava 20–30 seconds prior to blood drawing from the same vein into a syringe. Blood samples were centrifuged for 15 minutes at 3000 rpm at RT, subsequently plasma was centrifuged for 5 minutes at 13 000 rpm to remove remaining cells and platelets, and immediately frozen at -80°C.

##### Neutralizing heparin in plasma

Mice were anticoagulated by intravenous injection of 0, 4, 20 or 400 units heparin (Leo Pharmaceuticals, Weesp, The Netherlands), diluted in approximately 200 μl water for injection. After 5 minutes blood samples of approximately 700 μl were obtained by exsanguination via the vena cava into a syringe containing 0.1 volume 3.2% (w/v) sodium citrate. The plasma samples were centrifuged for 10 minutes at 3000 rpm and immediately frozen at -80°C until use. After thawing, a quarter of a heparinase tablet (Dade Hepzyme, Dade Behring, Marburg, Germany) was added to 150 μl of plasma sample in a plastic tube, mixed gently and incubated at room temperature for 15 minutes. Then, activated partial thromboplastin time (APTT) and factor VIII were measured with standard procedures with a Behring Coagulation System (BCS, Dade Behring) using human reagents as provided by the manufacturer. For factor VIII measurements human FVIII deficient plasma (Dade Behring) was used.

### Statistical analysis

Data are presented as mean with SEM. Differences between two groups were tested with students' t test. Differences between three groups were tested by one way ANOVA. P < 0.05 was set as threshold of statistical significance. All computations were performed using SPPS 11.0.

## Results

### Development of a mouse TAT-ELISA

#### Generation of rabbit antibodies against mouse thrombin and rat antithrombin

Rabbits were immunized with mouse thrombin or with rat antithrombin (AT). Before immunization neither of the rabbits exhibited immunoreactivity towards (xenogenic) thrombin and AT (data not shown). Twenty-five days after immunization the rabbits exhibited a response against the immunizing protein, which reached maximal levels 50 days after immunization. Serum immune titers of each rabbit were determined by ELISA. Serum samples of rabbits immunized with thrombin could be diluted more than 25,000-fold before the reactivity with thrombin reached background level (data not shown). Serum samples of rabbits immunized with AT could even be diluted 100,000-fold before reaching background signal (data not shown). Since antibodies from two rabbits recognized thrombin or antithrombin with equal immunoreactivity, sera from each pair of animals were pooled and used for further experiments (data not shown).

**Figure 1:**
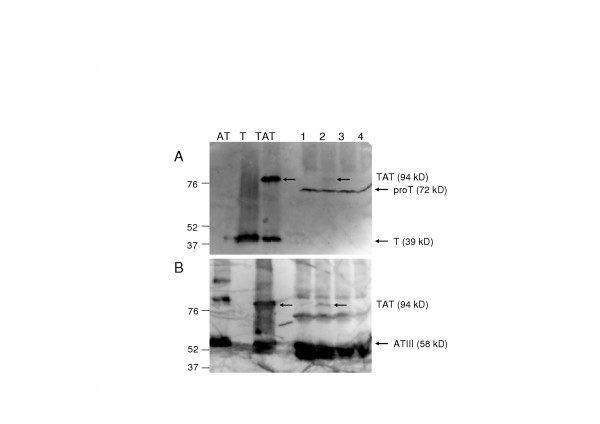
Antithrombin (lane AT), thrombin (lane T) and *in vitro *TAT complexes (lane TAT) were separated on a 7.5% acrylamide gel and transferred to a nitrocellulose filter. Plasma samples of control mice (lane 1) or mice treated with 50 μg/ml LPS for 2 (lane 2), 6 (lane 3) and 24 hours (lane 4) were separated also. The filters were incubated with 1000-fold diluted serum from a rabbit immunized with thrombin (A), or serum from a rabbit immunized with AT (B).

Western blot analysis showed that serum from rabbits immunized with thrombin, reacted only with thrombin (lane T) and not with antithrombin (lane AT) (Figure 1A). In the lane loaded with *in vitro *TAT complexes, two bands were observed of respectively 39 kD and 94 kD. The 39 kD band represents thrombin, whereas the 94 kD corresponds to the reported molecular weight of TAT complexes [7]. Besides prothrombin (72 kD), TAT-complexes were detected in plasma samples from LPS-treated mice (Figure 1A, lanes 1 through 4), indicating proper recognition of TAT-complexes in mouse plasma by the rabbit α-thrombin antibodies.

With serum obtained from rabbits immunized with mouse AT, opposite results were obtained. No band was detectable in the lane loaded with thrombin while a 58 kD band was detectable in the lane loaded with AT (Figure 1B). The two upper bands, also detected in the plasma samples, are multimeric complexes of AT. Again, two bands were detected in the TAT-lane: one band of 58 kD, representing uncomplexed AT, and a 94 kD TAT complex band. Western-blot analysis of plasmas obtained from LPS-treated mice showed the presence of TAT-complexes recognized by the rabbit α-AT antibodies (Figure 1B, lanes 1 through 4).

#### Development of TAT-complex sandwich ELISA

To discriminate and select between capture and conjugating antibody, protein-A purified rabbit α-thrombin antibodies and rabbit α-AT antibodies were conjugated to digoxigenin (DIG). In brief, using 96-wells plates coated with thrombin or AT it was confirmed that DIG was successfully conjugated to both antibodies (data not shown). Subsequently, 96-wells plates were coated with either rabbit α-thrombin antibodies or rabbit α-AT antibodies in a concentration of 1 μg/ml. Thereafter, the plates were incubated with two-fold serial dilutions of *in vitro *TAT-complexes. Plates coated with rabbit α-AT antibodies were subsequently incubated with serial dilutions of DIG-conjugated rabbit α-thrombin antibodies, whereas plates coated with rabbit α-thrombin antibodies were incubated with serial dilutions of DIG-conjugated rabbit α-AT antibodies.

**Figure 2:**
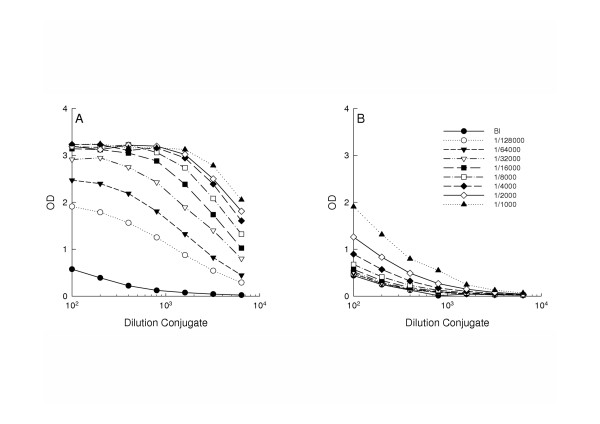
Selection of capture and conjugating antibody. 96-wells plates were coated with either rabbit anti-mouse thrombin antibodies (Panel A) or rabbit anti-rat antithrombin antibodies (Panel B) prior to incubation with two-fold serial dilutions of in vitro TAT-complexes. Plates coated with rabbit α-AT antibodies were subsequently incubated with serial dilutions of DIG-conjugated rabbit α-thrombin antibodies, whereas plates coated with rabbit α-thrombin antibodies were incubated with serial dilutions of DIG-conjugated rabbit α-AT antibodies.

The use of rabbit α-thrombin capture antibodies was superior to rabbit anti-rat antithrombin capture antibodies in combination with DIG-conjugated rabbit α-thrombin as detection antibodies in the detection of TAT-complexes, even at low concentration of secondary antibody (Figure 2A and 2B). Furthermore, the background signal was virtually zero in the absence of TAT-complexes and a 2000-fold dilution of DIG-conjugated rabbit α-AT antibodies. Detection of TAT-complexes using the combination of rabbit -AT antibodies as capture antibody and DIG-conjugated rabbit α-thrombin antibodies as detection antibody showed lower specificity compared to the reverse setup (Figure 2B). Therefore, for further experiments the rabbit α-thrombin antibodies and DIG-conjugated rabbit α-AT antibodies were used as capture antibody and detection antibody, respectively.

To further prove the possibility that positive results were caused by cross reactivity of rabbit α-thrombin antibodies with AT or vice versa, plates coated with rabbit α-thrombin antibodies were incubated with serial dilutions of thrombin or AT alone and thereafter incubated with DIG-conjugated rabbit α-AT antibodies. Neither thrombin nor AT alone were detected in this analysis (data not shown).

*In vitro *TAT-complexes were generated in order to be used as standard. The linearity of standard diluted in buffer was similar to the linearity upon dilution in mouse plasma or pooled human pool plasma (data not shown). While lower OD's were detected for standard diluted in human plasma as compared to buffer, the slopes of the linear part of the curves of standard diluted in human plasma (slope = 0.45, r^2 ^= 0.996) or buffer (slope = 0.43, r^2 ^= 0.998) were similar.

Using frozen aliquots of *in-vitro *TAT-complexes as standard, the intra- and inter-assay coefficients of variation (CV) of the ELISA were determined. The intra-assay CV were assessed by measuring TAT values in three samples each from different aliquots (n = 5) on one plate. The mean TAT concentration in the three samples was 5.1, 11.3 and 15.4 ng/ml and the corresponding CV were 7.7, 6.6 and 5.5 %. The three samples with different TAT levels were also analyzed on five separate ELISA plates and the inter-assay CV were 7.0, 5.1 and 4.7 %, respectively.

### Comparision of three TAT ELISA's in vitro

**Figure 3:**
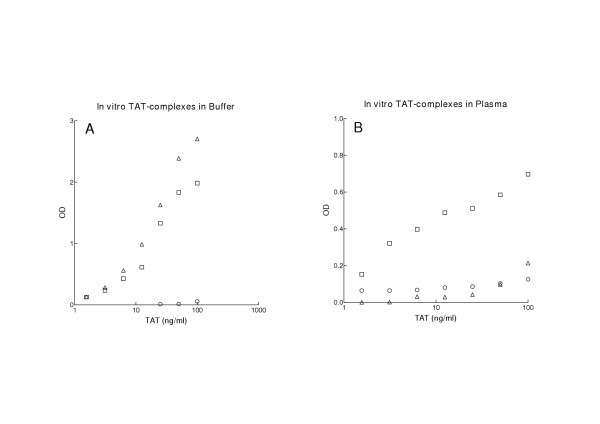
Comparison of three TAT ELISA's using in vitro TAT-complexes diluted in buffer (Panel A) or normal mouse pool plasma (Panel B). In vitro TAT-complexes were generated according to the method from Boisclair *et al*. (8). TAT-levels in serial dilutions of standards were measured by the mouse specific TAT ELISA (□), the human specific TAT ELISA from Enzyme Research Laboratories (△), and the human specific Enzygnost^® ^TAT micro from Dade Behring (○).

Detection of TAT-levels in standard prepared from *in vitro *TAT-complexes was compared between three ELISA methods: 1. the mouse specific TAT ELISA as described in Materials and Methods, 2. the human specific Enzygnost^® ^TAT micro from Dade Behring, and 3. the human specific TAT ELISA from ERL. Using the human specific TAT ELISA from ERL, a standard curve comparable to the mouse specific TAT ELISA was obtained by serial dilution of standard in buffer (Figure 3A). In contrast to the mouse specific ELISA, both commercial kits failed in detecting *in vitro *prepared TAT-complexes after serial dilutions in mouse plasma (Figure 3B).

### Plasma collection procedures

#### Anticoagulation with sodium citrate

**Figure 4:**
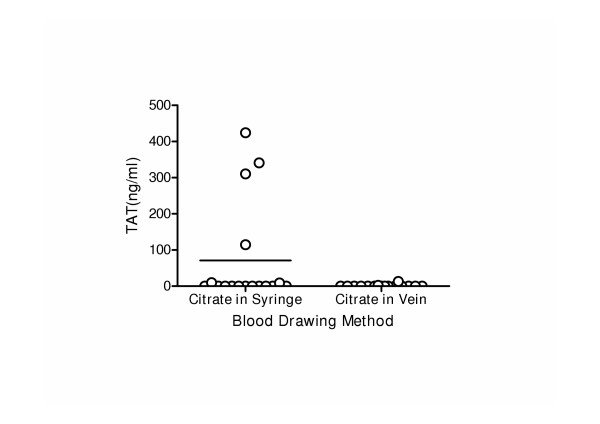
TAT-complex levels in mouse plasma after two different blood drawing techniques, determined with the home made TAT assay. Plasma samples were obtained by either collection of venous blood in a syringe filled with 180 μl 3.2 %(w/v) citrate (Citrate in Syringe) or by i.v. injection of 3.2% (w/v) sodium citrate (body weight (gr) / 13 × 10 μl) into the vena cave 20–30 seconds before blood drawing (Citrate in Vein).

Two different techniques of anticoagulation with sodium citrate were tested in mice. Collection of venous blood from the vena cava in a syringe with 0.1 volume sodium citrate resulted in activation of blood coagulation in four out of sixteen plasma samples, as indicated by high TAT-levels > 100 ng/ml, which indicated undesired activation (Figure 4). Plasma samples obtained by i.v. injection of sodium citrate (180 μl per mouse) via the vena cava showed little or no activation of blood coagulation, since fourteen samples had undetectable TAT-levels of 0 ng/ml and two with levels of 2 and 13 ng/ml (Figure 4).

#### Neutralizing heparin in plasma samples

**Figure 5:**
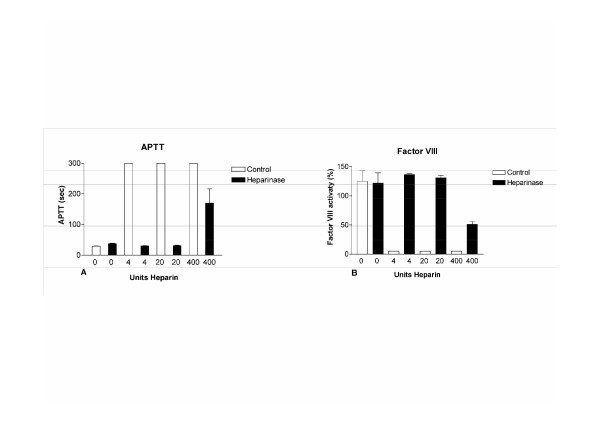
Neutralizing effect of heparinase on different amounts of heparin was tested. A) APTT returned to normal levels after heparinase addition to plasma samples from mouse treated with 4 and 20 units of heparin per mouse. B) Also, factor VIII activity returned to normal levels after heparinase treatment to plasma samples from mouse treated with 4 and 20 units of heparin.

To assess the influence of in vivo injected heparin and its neutralization with heparinase, APTT and factor VIII activity were measured. Intravenous injection of 4, 20 and 400 units of heparin prolonged APTT similarly in mouse plasma by 300 seconds. Neutralizing heparin through addition of heparinase shortened the APTT significantly (p < 0.0001) (Figure 5A). The APTT was the same (p = 0.6, tested by ANOVA) for plasma from mice treated with 4 or 20 units of heparin followed by heparinase neutralization compared to control plasma. The APTT of plasma pretreated with 400 U of heparin also shortened after heparinase addition, though it remained prolonged (169 seconds ± 47) compared to control plasma (29 seconds ± 1). Factor VIII activity was also normalized to normal levels after treatment with heparinase in plasma from mice treated with 4 or 20 units of heparin (p = 0.6, tested by ANOVA) (Figure 5B). The level of factor VIII in plasma fro mice treated with 400 units of heparin increased after heparinase addition (51 ± 5%) but not to the level of control plasma (125 ± 18%).

#### TAT measurement in vivo: the effects of LPS administration

**Figure 6:**
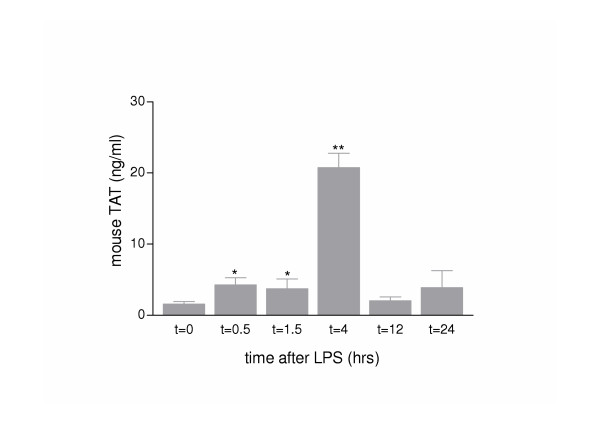
Increased TAT-complex levels in plasma of LPS-treated mice. Plasma samples were collected at 0, 0.5, 1.5, 4, 12, and 24 hours after i.p. injection of 2 mg/kg LPS. Values are means of triplicates ± SD. *; p < 0.05, and **; p < 0.0001.

To study the kinetics of TAT levels in plasma samples of endotoxin-treated mice and to evaluate the TAT ELISA, plasma samples were obtained at various time points after i.p. LPS administration. In plasma of control mice the TAT concentration was 1.6 ± 0.4 ng/ml (Figure 6). A 10-fold increase in TAT values up to 20.9 ± 2.0 ng/ml was observed in plasma samples obtained 4 hours after LPS administration. After 24 hours the TAT levels were back at baseline levels. The TAT levels on t = 0.5, t = 1.5 and t = 4 hours differed significantly from control levels (p < 0.05, unpaired Student' t-test).

**Figure 7:**
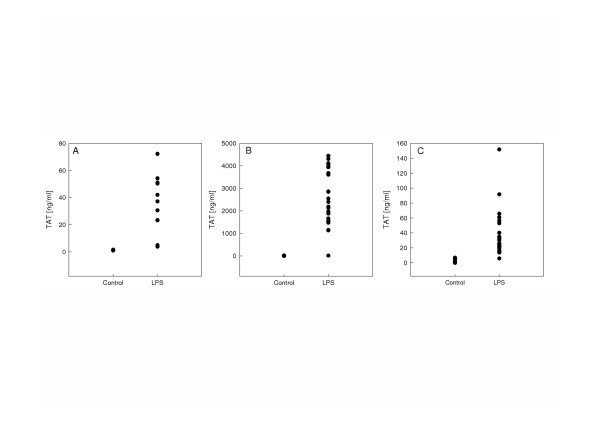
TAT-complex levels in mouse plasma without or after 6 hours of endotoxin treatment (2 mg/kg) as measured with the mouse specific TAT ELISA (panel A), the human specific TAT ELISA from Enzyme Research Laboratories (panel B), or the human specific Enzygnost^® ^TAT micro from Dade Behring (panel C). No correlation was found between TAT-concentration measured with our own ELISA and either the human specific TAT ELISA from Enzyme Research Laboratories (slope = 33.27, r^2 ^= 0.360), or the human specific Enzygnost^® ^TAT micro from Dade Behring (slope = 0.73, r^2 ^= 0.132).

It should be noted that the actual variation in peak TAT levels was very small in this experiment, which is in contrast with the observed range in values in the next experiment that was done at a later stage in different mice (Figure 7).

For comparison of *in vivo *formed TAT complexes in mouse plasma by the three different ELISA methods, the standards supplied in each commercial assay were diluted according to the manufactures instructions and used to calculate TAT-concentrations. Plasma samples were obtained from control mice and from mice six hours after i.p. LPS administration (2 m/kg) by drawing venous blood after i.v. administration of sodium citrate as described before. In plasma of control mice TAT concentration was 2.9 ± 2.9 ng/ml, 9.2 ± 7.7 ng/ml, and 1.1 ± 0.20 ng/ml, measured with respectively the Dade Behring, ERL, and our own ELISA (Figure 7). After 6 hours of endotoxemia blood coagulation was activated as indicated by a 30-fold increase in TAT-levels as measured with the mouse specific TAT ELISA (35.7 ± 21.1 ng/ml; p < 0.05 as compared to control plasma) (Figure 7), whereas TAT levels measured by the ERL and Dade Behring ELISA's were 2588.5 ± 1234.0 ng/ml and 37.4 ± 32.4 ng/ml, respectively. Of note, the response detected *ex vivo *by the three assays was quite comparable, whereas the *in vitro *comparison suggested that at least in plasma our TAT assay would be superior as compared to the commercial assays. We have no proper explanation for this discrepancy. Theoretically, a difference in affinity of the assay antibodies against the mouse-mouse TAT in *ex vivo *plasma as compared to the mouse-rat TAT complexes *in vitro *may play a role. Alternatively, the commercial assays could lack specificity for mouse TAT complexes, which could also explain the considerabble differences in TAT concentrations that are between 10–100 fold higher in the ERL assay as compared to our TAT method. We have no evidence to substantiate either of these explanantions, other than that a weak correlation, as measured with Spearman correlation test, between the mouse specific TAT ELISA and the human specific ELISA from ERL r = 0.600; p = 0.051 suggests that a similar product is being detected. No correlation was found between TAT-concentration measured with the mouse specific ELISA and the Dade Behring ELISA. A correlation was found between the two commercial human specific ELISAs (r = 0.694; p < 0.05).

## Discussion

Studying the relationship between coagulation changes in blood and at tissue level in mice requires the availability of tools for the analysis of coagulation activation. In this study we evaluated several procedures for plasma preparation and the measurement of coagulation activation in a mouse model of endotoxemia. First, we described the development of a specific mouse sandwich ELISA to measure the level of TAT complexes in mouse plasma. Second, we compared three different sandwich ELISAs for measurement of TAT levels in mouse plasma. Third, we tested different anticoagulation techniques to prevent ex vivo clotting.

Using the new TAT ELISA we detected changes in mouse plasma TAT content 4 hours after endotoxin infusion. Previously, it was demonstrated that endotoxin treatment of mice resulted in increased TAT complexes [8], TF mRNA synthesis [9] and enhanced tissue deposition of fibrin [10]. In human volunteers endotoxin infusion induced monocyte TF mRNA expression and plasma F1+2 and TAT levels [11]. Therefore, we expected an increase of TAT levels in plasma of mice treated with endotoxin. Indeed, basal TAT levels of 1.6 ± 0.4 ng/ml were increased in mice treated with endotoxin with maximal levels of 20.1 ± 9.9 ng/ml after 4 hours.

In addition to the mouse specific TAT ELISA, the two commercially available human TAT ELISAs, Enzygnost^® ^TAT micro from Dade Behring and the TAT ELISA from Enzyme Research Laboratories also detected increases in TAT levels after endotoxin treatment in mice. However, significant differences between the three assays were observed. The levels of TAT complexes as measured with the ERL assay were about 70 fold higher as compared to the two other assays. Furthermore, the Dade Behring assay could not detect *in vitro *prepared TAT complexes diluted in either buffer or plasma and *in vivo *TAT levels did not correlate to the levels measured with the mouse specific ELISA. Since plasma levels of TAT complexes measured by the ERL ELISA correlated to levels measured with the mouse specific ELISA, we speculate that this assay has probably a slightly better specificity for mouse TAT complexes than the Dade Behring assay. In addition, the ERL ELISA could measure *in vitro *prepared TAT complexes diluted in buffer. Still, the substantial variation in measured TAT concentrations suggests that the ERL assay, developed for use in human plasma, is not sufficiently specific for mouse TAT complexes. In additioon to these practical considerations and limitations, the considerable costs of the commercial human assays makes by comparison our novel assay to a cheaper and probably reliable alternative for the analysis of mouse TAT complexes in plasma samples.

For *ex vivo *anticoagulation sodium citrate is the most commonly used reagent added to human plasma. Since we experienced that anticoagulation with sodium citrate or EDTA in the syringe did not always effectively prevent *ex vivo *clotting of mouse blood, an alternative method of anticoagulation was sought. This led to a novel method to anticoagulate the blood samples in which sodium citrate was injected intravenously into the vena cava 20–30 seconds before blood collection by exsanguination. In our experiments, TAT levels were not increased in any of the plasma samples obtained using the i.v. injection of sodium citrate method, in contrast to the standard method in which sodium citrate was added to blood in the syringe. Therefore, we conclude that anticoagulation of mouse blood by i.v. injection of sodium citrate into the circulation results in more reliable TAT data and in plasma more suitable for further analysis of coagulation and related factors.

For immunohistochemical analyses of tissues pathologists may prefer the infusion of heparin prior to sacrificing the animal. To determine whether this anticoagulant effect could be eliminated to allow other assays including plasma based clotting tests, we tested the neutralizing activity of heparinase in mouse plasma. In accordance with results in human plasma we confirmed that up to 20 units of heparin per mouse, prolonging the APTT to more than 300 seconds, could be adequately neutralized with heparinase as indicated by normalized FVIII activity and APTT levels [6].

## Conclusion

In conclusion, the home made ELISA for mouse TAT complexes detects thrombin generation after endotoxin challenge in mice. Applied to blood samples obtained after systemic citrate infusion, this methodology may improve the quality of studies aimed at elucidating the pathophysiology of coagulation activation in mice.

## List of abbreviations

aPTT activated partial thromboplastin time

AT antithrombin

BSA bovine serum albumin

HRP horseradish peroxidase

LPS lipopolysaccharide

PBS phosphate buffered saline

RT room temperature

TAT Thrombin-Antithrombin complex

## Authors' contributions

Dirkje W. Sommeijer was involved in planning, experimental setup, development of methods, analysis of samples and writing the manuscript, René van Oerle was involved in murine blood sample collections, development of methods, and analysis of plasma samples, Pieter H. Reitsma was involved in experimental setup, analysis, and writing the manuscript, Janneke J. Timmerman was involved in design and development of antibodies against thrombin and antithrombin, Joost C.M. Meijers was involved in analysis, experimental setup, and writing of the manuscript, Henri M.H. Spronk was involved in animal work, development of methods, and writing of the manuscript, Hugo ten Cate was involved in planning, experimental setup, and writing of the manuscript.
